# HDL in diabetic nephropathy has less effect in endothelial repairing than diabetes without complications

**DOI:** 10.1186/s12944-016-0246-z

**Published:** 2016-04-14

**Authors:** Yufeng Li, Mingming Zhao, Dan He, Xuyang Zhao, Wenjing Zhang, Lixin Wei, Edgar Huang, Liang Ji, Meng Zhang, Belinda Willard, Zuodi Fu, Lijuan Wang, Bing Pan, Lemin Zheng, Linong Ji

**Affiliations:** Department of Endocrinology and Metabolism, Peking University People’s Hospital, No.11 Xizhimen Nan Dajie, Xicheng District, Beijing, 100044 China; The Institute of Cardiovascular Sciences and Institute of Systems Biomedicine, School of Basic Medical Sciences, and Key Laboratory of Molecular Cardiovascular Sciences of Ministry of Education, Peking University Health Science Center, No.38 Xueyuan Road, Haidian District, Beijing, 100191 China; Department of Obstetrics, The Military General Hospital of Beijing, Beijing, 100700 China; Department of Nephrology, Fujian Provincial Hospital, Fujian Medical University, Fuzhou, China; School of Informatics and Computing, Indiana University-Purdue University Indianapolis, Indianapolis, Indiana USA; Cleveland Clinic Lerner Research Institute Mass Spectrometry Laboratory for Protein Sequencing, Cleveland, Ohio USA; Department of Endocrinology and Metabolism, Capital Medical University Pinggu Teaching Hospital, Beijing, 101200 China

**Keywords:** High density lipoprotein, Cell migration, Glycation, Type 2 diabetes, Diabetic nephropathy

## Abstract

**Background:**

Diabetic nephropathy has a high cardiovascular risk with a low-level HDL(high density lipoprotein) in epidemiologic studies. Glycated HDL in diabetes can diminish the capacity to stimulate endothelial cell migration, but the mechanism has not been adequately explored in diabetic nephropathy. We performed this study to find out whether HDL in diabetic nephropathy is more dysfunctional than HDL in diabetes without complications.

**Methods:**

Endothelial cells were treated with N-HDL (normal), D-HDL (T2DM[type 2 diabetes mellitus] without complications), DN-HDL (T2DM nephropathy), N-apoA-I (normal apoA-I), and G-apoA-I (glycated apoA-I in vitro). Cell migration capacity was measured with wound-healing and transwell migration assay in vitro and electric carotid injury model in vivo. Protein glycation levels were measured with nanoLC-MS/MS. PI3K expression and Akt phosphorylation were analyzed by western blot.

**Results:**

In wound-healing assay, DN-HDL showed a 17.12 % decrease compared with D-HDL (*p* < 0.05). DN-HDL showed a 29.85 % decrease in comparison with D-HDL (*p* < 0.001) in transwell assay. In the electric carotid injury model, D-HDL and DN-HDL impaired the re-endothelialization capacity; DN-HDL was less effective than D-HDL. Meanwhile, DN-HDL was found to have a significantly higher protein glycation level than D-HDL (*p* < 0.001). PI3K expression and Akt phosphorylation were reduced significantly in DN-HDL in comparison with D-HDL and N-HDL.

**Conclusions:**

We found that HDL from diabetic nephropathy has a higher level of glycation and induced less cell migration in vitro and in vivo compared with that from diabetes without nephropathy. This finding suggests that diabetic nephropathy has higher levels of glycated HDL and partially explains why patients with DN have a higher risk of cardiovascular disease.

**Electronic supplementary material:**

The online version of this article (doi:10.1186/s12944-016-0246-z) contains supplementary material, which is available to authorized users.

## Background

Type 2 diabetes mellitus (T2DM) and chronic kidney disease (CKD) are two major growing public health problems all over the world [1, 2]. Cardiovascular disease is the leading cause of morbidity and mortality of diabetes, accounting for up to 80 % of premature mortality in diabetes patients [3, 4]. Many epidemiological and clinical trials have demonstrated that diabetes is the major cause of CKD with the risk of developing CVD(cardiovascular disease) when CKD and diabetes coexist [1, 5, 6]. However, why there is higher cardiovascular risk when diabetes coexists with CKD is not clear [3].

Epidemiological studies have provided overwhelming evidence that arteriosclerosis is core to the pathological process of cardiovascular diseases with dyslipidemia being an important risk factor for atherosclerosis in diabetes [7, 8]. High levels of LDL(low density lipoprotein) and low levels of HDL are typical features of dyslipidemia and both have been shown to be independent predictors of CVD in diabetes [9–14]. Additionally, a prospective study showed that low baseline HDL levels are a significant and independent predictor of the development and progression of diabetic nephropathy (DN) [15].

The main component of HDL is apolipoprotein A-I (apoA-I) [16, 17]. HDL can enhance endothelial progenitor cell endothelium repair and stimulate endothelia cell proliferation and migration by activating endothelial nitric oxide synthase [18, 19]. Endothelial damage is critical in the development of CVD, and endothelial cell migration is a rate-limiting process in the repair of endothelium [7]. HDL in diabetes is dysfunctional in stimulating endothelial cell migration and proliferation with glycation of HDL, one of contributors to its dysfunction [20, 21]. Dysfunctional HDL could be an important factor in the development of diabetic cardiovascular disease [16]. The relationship between HDL in DN and CVD has not been sufficiently explored and specifically whether there is a difference in HDL glycation between patients with DN and diabetes without complications. It has been demonstrated that HDL can interact with SR-BI and activate the small G protein Rac via Src kinase, phosphoinositol 3-kinase (PI3K), Akt, and ERK, which stimulate the rapid initial lamellipodia formation, an indicator of cell migration [7].

We hypothesized that the function of HDL is more compromised in DN patients than in diabetic patients without complications due to glycation, and that dysfunctional HDL leads to severely reduced capacity to stimulate endothelial cell migration. Using wound-healing assay, transwell migration assay in vitro, and electric injury model in vivo we investigated whether N-HDL (normal), D-HDL (T2DM without complications), and DN-HDL (T2DM nephropathy) have significantly different functions in stimulating EC migration. We explored the relationship between HDL glycation levels through mass spectrometry and the capacity to stimulate human umbilical vein endothelial cell (HUVEC) migration. We also attempted to find out whether these differences involved the PI3K/Akt pathways.

We found that HDL from DN has a higher level of glycation and induced less cell migration capacity compared with diabetes without complications. The mechanism of endothelial cell migration reduction partially explains why DN has a higher risk of cardiovascular disease.

## Methods

### Patient characteristics

Healthy volunteers (*n* = 12) and patients with type 2 diabetes mellitus without complications (*n* = 18) and patients with diabetic nephropathy without CVD (*n* = 18) were recruited from Capital Medical University Pinggu Teaching Hospital. Written informed consent was obtained from every participant before the study began, and the hospital’s ethics committee approved the protocol for data collecting involving human subjects for this study. Each volunteer went through a medical-history check, a physical examination, a 75 g oral glucose tolerance test, and other laboratory screening tests. Type 2 diabetes and diabetic nephropathy patients were all diagnosed by physicians. Type 2 diabetes patients without complications, which include nephropathy, retinopathy, CVD, and other vascular diseases, were enrolled. Diabetic nephropathy was defined as diabetes with the consistent presence of albuminuria (the ratio between urine albumin and the creatinine was 30 mg/g or higher [22]) or impaired GFR(glomerular filtration rate) or both. In addition, all participants in this group must have been diagnosed with diabetic retinopathy. About two-third (11 in total) diabetic and equivalent (12 in total) diabetic nephropathic patients were on antihypertensive, hypoglycemic and lipid-lowering medications. Clinical and laboratory characteristics of the study participants are shown in the Table 1.

**Table 1:** Patient characteristics

Characteristic	Healthy controls (*n* = 12)	Patients with diabetes (*n* = 18)	Patients with diabetic nephropathy (*n* = 18)
Age (years)	31.92 ± 2.11	57.78 ± 2.44***	59.94 ± 1.67
Diabetes duration(years)	0	5.18 ± 1.77	11.83 ± 1.63^#^
Fasting glucose (mmol/l)	4.64 ± 0.09	11.28 ± 1.15***	10.19 ± 1.24
Blood urea nitrogen (mmol/l)	4.55 ± 0.37	5.51 ± 0.41	8.15 ± 0.62^##^
HbA1C (%)	5.01 ± 0.12	10.04 ± 0.47***	8.91 ± 0.61
Creatinine (umol/l)	57.78 ± 4.70	57.61 ± 2.26	112.40 ± 15.49^##^
eGFR (ml/min/1.73 m2)	131.30 ± 8.42	131.10 ± 11.01	70.18 ± 5.33^###^
Total cholesterol (mmol/l)	4.25 ± 0.17	4.71 ± 0.21	4.76 ± 0.41
Triglycerides (mmol/l)	1.04 ± 0.10	2.51 ± 0.32**	1.89 ± 0.30
HDL-C (mmol/l)	1.47 ± 0.07	1.01 ± 0.05***	1.03 ± 0.08
LDL-C (mmol/l)	1.95 ± 0.10	2.90 ± 0.27**	3.02 ± 0.33

Data are expressed as mean ± SEM. *HDL-C* high density lipoprotein cholesterol, *LDL-C* low density lipoprotein cholesterol; **p<0.01, ***p<0.001, DM-HDL versus N-HDL; ^##^ *p*<0.01, ^###^ *p*<0.001, DKD-HDL versus DM-HDL, one-way ANOVA

### Animals

Six to eight-week-old male ICR mice were obtained from the Department of Laboratory Animal Science, Peking University, and the Ethics Committee of Animal Research, Peking University Health Science Center approved all animal experimental procedures.

### Cell culture

HUVECs were isolated by collagenase digestion of umbilical veins from fresh cords and cultured with endothelial cell medium containing 5 % bovine serum, 1 % endothelial cell growth supplement, and 1 % penicillin/streptomycin solution, in a humidified atmosphere (5 % CO2) at 37 °C. HUVECs used in all experiments were at passages 3–5. Cells were starved overnight for further treatment.

### Isolation of high-density lipoprotein

Fasting peripheral blood was collected in vacuum blood tube containing EDTA from healthy (N), diabetic (D) and diabetic nephropathy (DN) subjects. LDL (1.019–1.063 g/ml) and HDL (1.063–1.210 g/ml) were isolated from fresh plasma by ultracentrifugation after the plasma from 18 individuals was mixed as a pool [23]. The plasma was centrifuged at 550,000 g for 5 h at 4 °C. HDL was dialyzed against endotoxin-free phosphate-buffered saline (10 mM, PH7.4) for 3 days in the dark at 4 °C. The purity of HDL was confirmed by the 12 % sodium dodecyl sulfate polyacrylamide gel electrophoresis (SDS-PAGE). The concentration of HDL was measured by nephelometry (Dimension XPand, Dade Behring, Germany). HDL was sterilized through the 0.22 um filter and then stored in sealed tubes at 4 °C in the dark, ready to be used within 1 month.

### Endothelial cell migration assays

#### Wound-healing migration assay

Cell migration capacity can be reflected through wound-healing migration assay [24]. In this experiment, HUVECs were planted in 6-well plates with 1.2 ml endothelial cell medium containing 5 % bovine serum and cultured until monolayers were formed. Then HUVECs were scratched with a 200 ul micropipette tip, and cells were incubated with endothelial cell medium containing 1 % bovine serum respectively with PBS, N-HDL, D-HDL, or DN-HDL with an apoA-I concentration of 100 μg/ml for 10 h. Cells were photographed with an inverted microscope (Nikon, Japan), and cell migration was quantified using the migrated gap distance in six random high-power (50X) fields.

#### Transwell migration assay

Apart from wound-healing migration assay, quantitative cell migration assay was performed with a modified Boyden chamber (Minicell, Millipore, USA) with 8.0 um pore polycarbonate filter inserted in 24-well plates. HUVECs (4 × 10^5^cells/well) in endothelial cell medium without bovine serum were plated into the upper chamber. The lower chamber was filled with 500 ul endothelial cell medium containing 5 % bovine serum. HDL with an apoA-I concentration of 100 μg/ml were respectively co-cultured with HUVECs for 10 h. Then all non-migrated cells in the upper chamber were removed with a cotton swab. Migrated cells were fixed and stained with 0.1 % (M/V) crystal violet. Cells in each chamber were photographed with an inverted microscope (Nikon, Japan). Cell migration was quantified by counting the number of stained cells per field in six random high-power (50X) fields.

### Glycation analysis by mass spectrometry

HDL (30 ug protein per lane) was subjected to electrophoresis on 10 % SDS-polyacrylamide gels (SDS-PAGE). The specific gel band (apoA-I) was excised and destained with 25 mM NH4HCO3 in 50 % acetonitrile. Proteins were reduced by 10 mM Dithiothreitol and alkylated by 50 mM Iodoacetamide. After being dried in 100 % acetonitrile, the gel band was digested using sequencing grade trypsin (Promega) at 37 °C overnight. The extracted peptides were suspended in 0.1 % formic acid and subjected to nanoLC-MS/MS analysis. Peptides were eluted with a linear gradient from 5 to 40 % of 100 % acetonitrile and 0.1 % formic acid at a flow rate of 300 nl/min using a 100 μm * 10 cm reversed-phase C18 fused silica emitter made in house. The data-dependent mass spectra were acquired with LTQ Orbitrap Elite mass spectrometer (Thermo Fisher Scientific) equipped with a nanoelectrospray ion source (Thermo Fisher Scientific). Raw mass spectra files were processed with Proteome Discoverer 1.4 (Thermo Fisher Scientific) and searched in the human Uniprot database (version 2014_02) through the SEQUEST search engine. The precursor ion mass tolerance was set to 10 ppm, and MS/MS tolerance 0.02 Da. Searching parameters were set as follows: trypsin up to two missed cleavages, carbamidomethyl cysteine as fixed modification, methionine oxidation and glycation of lysine as variable modifications.

### Electric injury model

Cell migration capacity in vivo was measured through the electric injury model [25]. In general, mice were anesthetized with 10 % (M/V) chloral hydrate at a dose of 3 ml/kg by intraperitoneal injection. Then the head and the limbs were fixed after about 10 min. Neck skin was sterilized with alcohol. Blunt dissect was performed on the skin under a stereoscopic microscope (CNMICRO, SMZ-B2), and then the left carotid artery was isolated. A 4 mm plastic gasket was placed under the left carotid artery in order to avoid damage to other tissues. An electric current of 0.8 mA was applied for 2 s three times. N-HDL, D-HDL, DN-HDL with an apoA-I content of 100 ug respectively were injected through the tail vein every other day after carotid artery was injured. On the first, third, and seventh day after carotid artery injury, mice were anesthetized, and the injured vessel segments were dissected and fixed in 4 % formalin for 8 h and then transferred into 20 %(M/V) sucrose solutions overnight. Afterwards, vessel segments were embedded under the optimal cutting temperature (ZLI-9302, Zhongshan Goldenbridge Biotechnology Co., Ltd), sharp-frozen in liquid nitrogen for 20 min, and stored in -80 °C refrigerator for further use. Vessel segments were cut into 7 um section with a freezing microtome (Leica, Germany), and endothelial cell was stained with hematoxylin–eosin, rabbit anti-CD31 antibody (ZA-0568, Zhongshan Goldenbridge Biotechnology Co., Ltd). Cell proliferation was detected with mouse anti-PCNA (proliferating cell nuclear antigen) antibody (ZM-0213, Zhongshan Goldenbridge Biotechnology Co., Ltd) for immunohistochemistry.

### Western blot

The expression of PI3K and p-Akt were analyzed by Western blot [21]. HUVECs were cultured in 6-well and starved overnight until monolayers were formed. Cells were then incubated with serum-deprived endothelial cell medium respectively with PBS, N-HDL, D-HDL, or DN-HDL with an apoA-I concentration of 100 μg/ml for 15 min. After the treatment, cells were harvested and lysed with 100 ul RIPA (C1053, Beijing Applygen Technologies Inc.) containing 1 ul PMSF (P0100, Beijing Solarbio Science & Technology Co., Ltd). Cell debris was removed by centrifugation at 12,000 rpm for 20 min, and protein concentration was determined with the Coomassie brilliant blue method. Cell lysates (60 ug protein per lane) were subjected to electrophoresis on 10 % SDS-polyacrylamide gels (SDS-PAGE) and transferred onto nitrocellulose membranes (Pall Corporation, USA) according to standard procedures. Then the membranes were blocked for 2 h with 5 % non-fat milk. Membranes were incubated with each primary antibody (1:500–1:1000 dilution) overnight at 4 °C followed by the appropriate horseradish peroxidase (HRP)-conjugated secondary antibody (1:1000 dilution). The expression was detected using the Super Signal West Pico Kit (Pierce, USA) based on the manufacturer’s instructions.

### Statistical analysis

The results of multiple observations are presented as the means ± SEM. Data were analyzed with one-way ANOVA or two-tailed Student’s t-test using GraphPad Prism software (GraphPad Prism Software, USA). Any difference with *p* < 0.05 was considered statistically significant. ANOVA was used to detect the differences among three or more groups. If p was smaller than 0.05, a post-hoc test was used to determine the differences between groups for further analysis.

## Results

### Diabetic nephropathy high-density lipoprotein has a decreased capacity to stimulate HUVECs migration compared to diabetic high-density lipoprotein

**Fig. 1:**
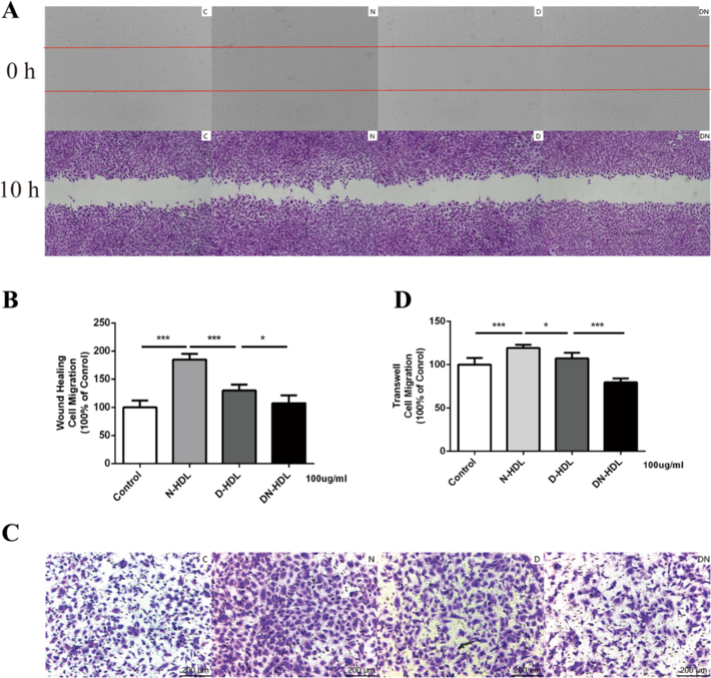
Diabetic HDL and diabetic nephropathy HDL are less efficient in promoting HUVEC migration. **a** HUVEC monolayers were scratched by manual scraping and treated with PBS(C), N-HDL (N), D-HDL (D), or DN-HDL (DN) respectively at 100 ug/ml apoA-I for 10 h. Migration into the wound was photographed (50X objective lens). The red line indicated the scratch edge. **b** Distance between gaps was measured, and the results were expressed as percentage of various HDL-treated cells in comparison with control. **c** HUVECs were treated with PBS(C), N-HDL (N), D-HDL (D), or DN-HDL (DN) at 100 ug/ml apoA-I for 10 h respectively in transwell assay, and pictures were taken in six random high-power (50X) fields. **d** Migratory cells were counted, and the results were expressed as percentage of various HDL-treated cells in comparison with control. Each experiment was conducted in duplicate in three repeats and HDL were pooled from six individuals with different patients in each repeat. (*n* = 12 in control group, *n* = 18 in diabetes group, and *n* = 18 in diabetic nephropathy group, **p < 0.01; ***p < 0.001; one-way ANOVA)

HUVECs migration capacity was measured through wound-healing migration assay and transwell migration assay in vitro. In wound-healing assay, DN-HDL showed a 17.12 % decrease compared with D-HDL (*p* < 0.05), and 32.2 % decrease of D-HDL to N-HDL (*p* < 0.001, Fig. 1a and b). To conform the effects on cells migration, transwell assay was carried out and DN-HDL showed a 29.85 % decrease in comparison with D-HDL (*p* < 0.001). D-HDL showed a 9.9 % decrease in comparison with N-HDL (*p* < 0.01, Fig. 1c and d). Taken together, N-HDL promoted the migration of EC, while D-HDL and DN-HDL had a decreasing function, and the decrease was severe in DN-HDL.

### D-HDL and DN-HDL have impaired re-endothelialization capacity in vivo

**Fig. 2:**
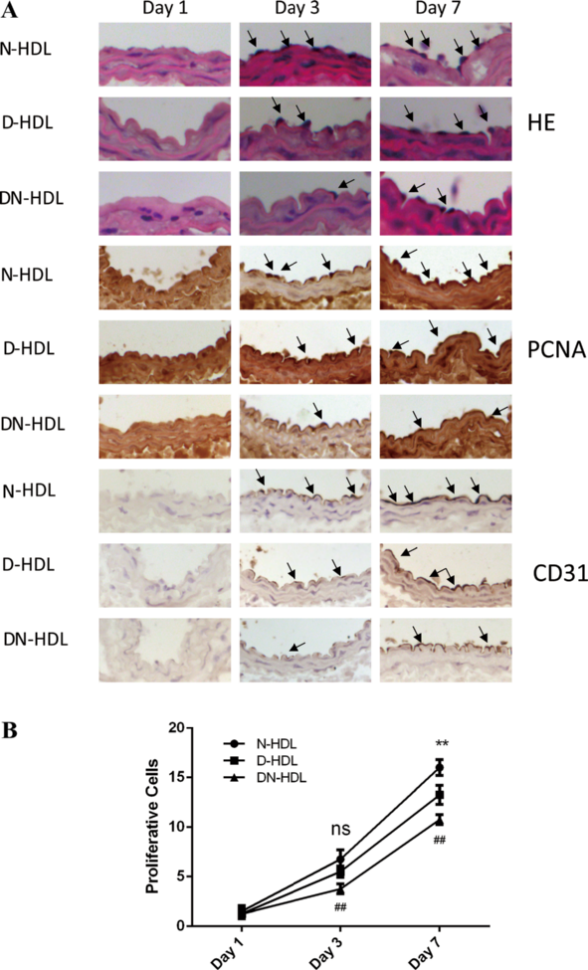
D-HDL and DN-HDL have a reduced capability to promote reendothelialization in the electric injury model in vivo. **a** Frozen sections of injured arteries on days 1, 3, and 7 treated with N-HDL, D-HDL and DN-HDL are stained with HE (hematoxylin–eosin), PCNA and CD31. Bar, 20 μm. **b** Quantitative analysis of cells in neointima was conducted using Prism (*n* = 4/group, **p < 0.01, D-HDL versus N-HDL; ^##^ *p* < 0.01, DN-HDL versus D-HDL; one-way ANOVA)

The re-endothelialization capacity of endothelial cells in vivo was measured using an electric carotid injury model [25]. Reendothelialization is the repopulation of the intima impaired by endothelia cell migration from the injury border [26]. Endothelial cells were delineated using CD31 antibody and standard hematoxylin–eosin staining. Proliferating endothelial cells were identified by being stained with proliferating cell nuclear antigen (PCNA) antibody [27] (Fig. 2a). On Day 1, migration cells were barely observable. On Day 3, cell quantity increased quickly when treated with N-HDL compared with Day 1 (*p* < 0.001). Cells numbers on day 7 were higher than on Day 3 (*p* < 0.001). Cell migration increased at the injury section in a time-dependent manner. In comparison with D-HDL, DN-HDL showed a 31.82 % decrease (*p* < 0.01) in cell migration on Day 3 while N-HDL and D-HDL had no difference. On Day 7, DN-HDL showed an 18.87 % decrease compared with D-HDL (*p* < 0.01), and D-HDL decreased 17.19 % in comparison with N-HDL (*p* < 0.01) (Fig. 2b).

### Diabetic and diabetic nephropathy high density lipoprotein have a high level glycation level through HPLC/MS/MS

**Fig. 3:**
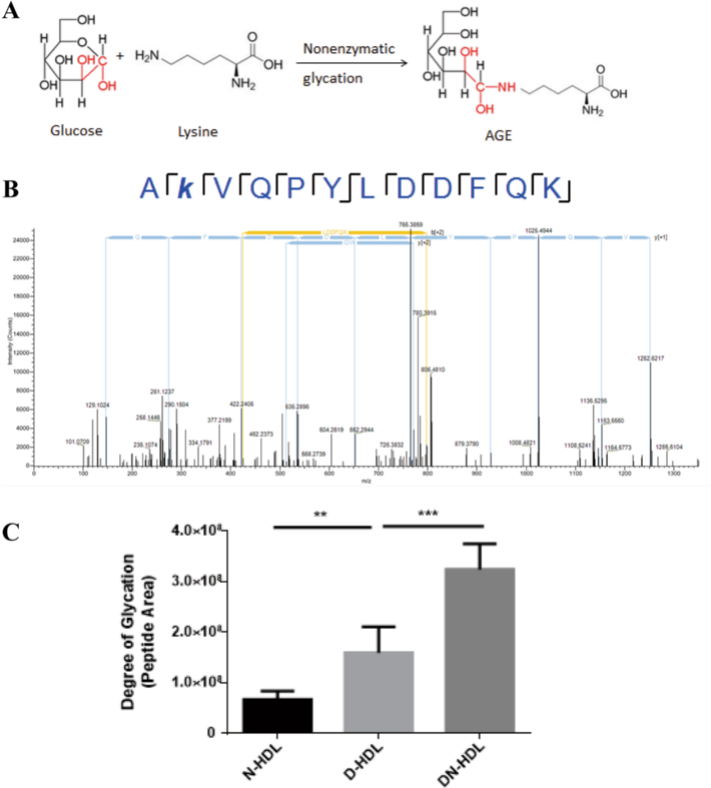
Diabetic and diabetic nephropathic high-density lipoprotein have a high glycation level. **a** ApoA-I gel bands are digested and analyzed by HPLC/MS/MS of N-HDL, D-HDL and DN-HDL samples. A: schematic diagram of lysine glycation with a molecule glucose. **b** The CID spectra of A*k*VQPYLDDFQK specifically identify glucose modification that occurs on K96. **c** The peak areas for the modified peptide in three groups. Results are presented with mean ± SEM in six separate experiments (*n* = 6/group, **p < 0.01,***p < 0.001, one-way ANOVA)

Advanced glycation end products (AGEs) are the nonenzymatic reaction of glucose and other reducing sugars with amino groups of proteins. N-(Carboxymethyl)lysine (CML) and N-(Carboxyethyl)lysine (CEL) are two major nonenzymatic chemical modifications to proteins [28]. Except for CML and CEL, a new type of AGEs in which lysine was modified with a molecule of glucose was found in our experiment (Fig. 3a). An example MS/MS spectrum is shown in Fig. 3b. This spectrum was derived from the [M + H] + 2 form of the Ak162VQPYLDDFQK peptide. Five glycated lysine residues in apoA-I were monitored. DN-HDL showed a 2.55-fold-level increase compared with D-HDL (DN-HDL 3.24e + 008 VS D-HDL 1.27e + 008, *p* < 0.001), and D-HDL showed a 1.63-fold-level increase compared with N-HDL (D-HDL 1.27e + 008 VS N-HDL 7.08e + 007, *p* < 0.01) (Fig. 3c).

### Glycated apoA-I (G-apoA-I) and glycated HDL (G-HDL) have a reduced capacity to stimulate endothelial cell migration

**Fig. 4:**
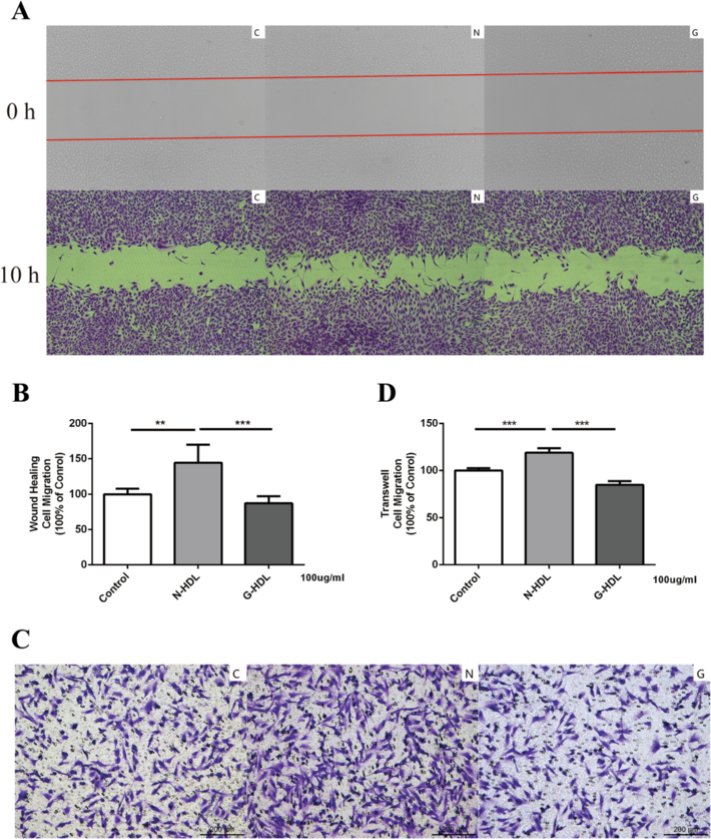
Glycated HDL is less efficient in promoting HUVEC migration than normal HDL. **a** HUVEC monolayers were manually scratched and treated with PBS(C), N-HDL (N), G-HDL (G) at the 100 ug/ml apoA-I for 10 h. Migration into the wound was photographed (50X objective lens). The red line indicated the scratch edge. **b** Distance between gaps was measured, and the results were expressed as percentage of various HDL-treated cells in comparison with control. **c** HUVECs were treated with PBS(C), N-HDL (N), or G-HDL (G) respectively at 100 ug/ml apoA-I for 10 h in transwell assay. **d** Migratory cells were counted, and the results were expressed as percentages of various HDL -treated cells in comparison with control (**p < 0.01; ***p < 0.001; one-way ANOVA)

**Fig. 5:**
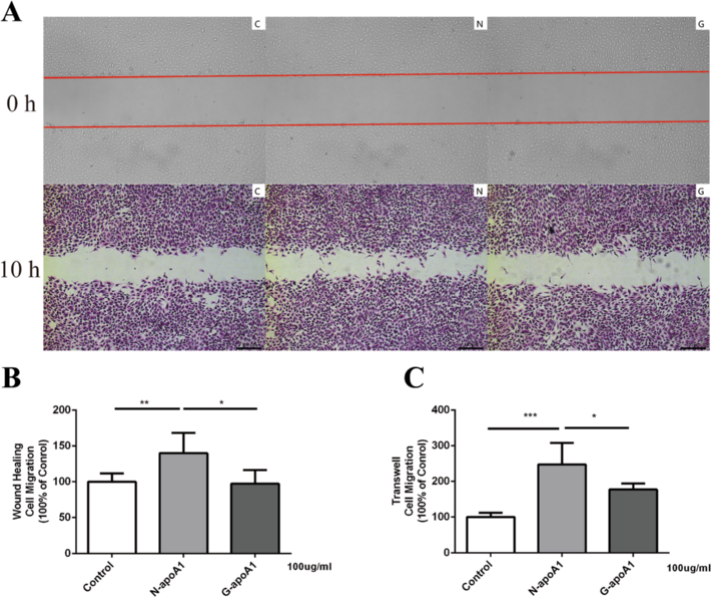
Glycated apoA-I is less efficient in promoting HUVEC migration compared with normal apoA-I. **a** HUVEC monolayers were manually scratched and treated with PBS(C), N-apoA-I (N), G-apoA-I (G) at the 100 ug/ml apoA-I for 10 h. Migration into the wound was photographed (50X objective lens). The red line indicated the scratch edge. **b** Distance between gaps was measured, and the results were expressed as percentages of various apoA-I-treated cells in comparison with control. **c** HUVECs were treated with PBS(C), N-apoA-I (N), or G-apoA-I (G) respectively at 100 ug/ml apoA-I for 10 h in transwell assay. Migratory cells were counted, and the results were expressed as percentages of various apoA-I -treated cells in comparison with control (*p < 0.05;**p < 0.01; ***p < 0.001; one-way ANOVA)

HDL is a complex mixture of lipoproteins associated with many minor proteins and lipids which influence HDL function [29, 30]. ApoA-I is the major component in HDL, and many studies have found apoA-I can be modified in a disease state damaging the apoA-I function [31, 32]. In order to find out whether HDL glycation causes HDL dysfunction and eliminate the impact of other ingredients in HDL except for apoA-I, normal HDL and apoA-I were incubated in 25 mM glucose solution at 37 °C over 7 days. HDL glycation level was confirmed using HPLC/MS/MS (Additional file 1: Figure S1). Then glycated HDL (G-HDL) and glycated apoA-I (G-apoA-I) were used to measure the migration capacity through wound-healing assay and transwell migration assay. G-HDL showed a 39.58 % decrease compared with N-HDL (G-HDL 87.25 ± 4.17 vs. N-HDL 144.40 ± 10.47, *p* < 0.001, Fig. 4a and b) in wound-healing assay. G-HDL was less effective in EC migration compared with N-HDL (G-HDL 84.78 ± 1.66 vs. N-HDL 118.90 ± 1.95, *p* < 0.001, 100 % of control, Fig. 4c and d) in transwell migration assay. G-apoA-I showed a 30.58 % decrease compared with N-apoA-I (G-apoA-I 97.19 ± 7.81 vs. N-apoA-I 140.00 ± 11.56, *p* < 0.05, Fig. 5a and b) in wound-healing assay and 28.42 % decrease (G-apoA-I 177.30 ± 6.69 vs. N-apoA-I 247.70 ± 24.65, *p* < 0.05, 100 % of control, Fig. 5c) in transwell assay. Thus, the function loss of D-HDL and DN-HDL in mediating cell migration is consistent with the apoA-I glycation.

### D-HDL and DN-HDL reduce endothelial cell migration involved in PI3K/Akt pathways

**Fig. 6:**
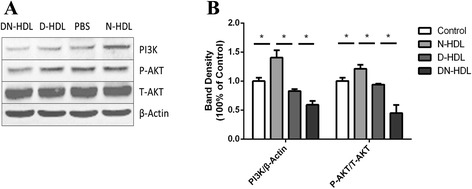
D-HDL and DN-HDL have less capacity to induce PI3K expression and Akt phosphorylation compared to N-HDL. **a** HUVECs were treated with PBS, N-HDL, D-HDL or DN-HDL respectively, at 100 ug/ml apoA-I for 15 min. Cell lysates were analyzed by Western blotting using anti-PI3K p85αantibody, anti-phospho-Akt(Ser473) antibody and anti-total-Akt antibody. **b** The density of the phospho-Akt bands and PI3K p85αbands were normalized to total-Akt and β-actin, respectively. (*p < 0.05; one-way ANOVA)

Cell migration involves the coordination of several signal-transduction pathways, including PI3K expression and Akt phosphorylation [7]. In order to illuminate the mechanisms with which D-HDL and DN-HDL inhabited cell migration, HUVECs were treated with PBS, N-HDL, D-HDL or DN-HDL respectively at 100 ug/ml apoA-I for 15 min. PI3K expression and Akt phosphorylation level were analyzed using specific antibody by Western blot. Compared with N-HDL, D-HDL and DN-HDL reduced the capacity to activate PI3K and Akt phosphorylation after 15 min of incubation with HUVECs (Fig. 6).

## Discussion

Clinical epidemiology has demonstrated that patients with DN have a high cardiovascular risk, and CVD has been identified as the primary cause of deaths in patients with DN [33]. Dyslipidemia exacerbates the pathogenesis of both CVD and DN [8]. Diabetic HDL is dysfunctional in stimulating endothelial cell migration due to down regulation of scavenger receptor B1 (SR-B1) expression [21]. We used wound-healing and transwell migration assays to assess the differences of HUVECs migration promoted by N-HDL, D-HDL and DN-HDL respectively showing that DN-HDL reduced the HUVECs migration capacity much more than D-HDL did in vitro and in vivo. A recent study indicates HDL lost its association with lower mortality in patients with even minor impairment of kidney function, whereas higher HDL levels almost turned into a potential cardiovascular risk factor in patients with more advanced kidney failure [34]. This is an important finding because DN is accompanied by an increased triglyceride and decreased HDL levels and we now know HDL disordered function in DN except its low levels suggesting a possible explanation for why DN has a higher risk of cardiovascular disease.

HDL is a complex mixture of lipoproteins associated with many minor proteins and lipids that influence HDL function [30]. ApoA-I constitutes 70 % of total HDL protein, which exerts primary HDL anti-atherosclerotic effects through RCT [32]. In order to eliminate the influence from such complexity, we used purified normal apoA-I glycated in glucose, and we found that the glycated apoA-I had a reduced capacity in endothelial cell migration compared with normal apoA-I (Figs. 4 and 5). As a result, glycated apoA-I in HDL accounts for the decreased migration capacity in HUVECs.

The formation and accumulation of advanced glycation end products (AGEs) are two of the most important mechanisms involved in the pathophysiology of chronic diabetic complications [35]. HDL is nonenzymatically glycated at an increased level in diabetic individuals, but little is known about the association between glycated HDL and endothelium dysfunction in diabetes [36]. We used mass spectrometry to analyze the level of glycated apoA-I, and the level could represent HDL glycation in vivo. We found HDL glycation levels were more salient in DN than in diabetes without complications (Fig. 3). The higher HDL glycation level, the further capacity to stimulate endothelial cell migration was reduced. A previous study indicated that apoA-I glycation level is affected by glucose concentration in vitro [16], and with a decrease in renal function, the clearance capacity of AGEs is diminished [37]. In this study, patients with DN had similar age and glucose levels but lower eGFR(estimated glomerular filtration rate) compared with those without nephropathy. These differences may contribute to the high level of glycated HDL in DN.

Oxidized HDL diminishes HUVEC migration through the PI3K/Akt and MEK/ERK pathways [38], and HDL activates cyclin D1 via phosphatidylinositol 3-kinase (PI3K)/Akt stimulation in the healing process by promoting EPC proliferation, migration and ‘tube’ formation [39]. To examine the mechanism in which DN-HDL and D-HDL reduce HUVECs migration in this study, we analyzed the expression of PI3K and p-Akt and found that N-HDL induced PI3K expression and Akt phosphorylation while D-HDL and DN-HDL had less capacity to activate these phosphorylations. In fact, the capacity reduction turned more severe in DN-HDL (Fig. 6).

The study used mass spectrometry to measure the level of HDL glycation both in diabetes without complications and DN and found that apoA-I glycation levels are higher in DN-HDL than in D-HDL, and that DN-HDL severely diminished cell migration capacity. It has been demonstrated that endothelial cell migration is the major mechanism of initial adjacent surface coverage in vitro [40]. Accordingly, delayed re-endothelialization due to inhibition of migration may contribute significantly to the formation of irreversible vascular disease [40]. HDL’s capacity to promote cell migration is one of the mechanisms for protecting vascular vessels from damages. Our findings suggest that glycation impairs HDL function, and dysfunctional HDL may lead to the reduction of the re-endothelialization capability and potentially explains why there is excessive CVD in DN patients.

## Conclusions

This study investigated HDL function in diabetic nephropathy and found that, compared with D-HDL, DN-HDL more significantly reduced the capacity to stimulate cell migration in vitro and in vivo due to a higher glycation level. These findings indicate one of the potential mechanisms that explain why diabetic nephropathy has a higher risk of cardiovascular disease. More attention should be paid to the level of HDL glycation and its function on CVD in diabetic nephropathy.

## Additional file

Additional file 1: Supplement Figure 1: Diabetic and diabetic nephropathic high-density lipoprotein is less efficient in stimulating EC proliferation. HUVECs were treated with N-HDL, D-HDL and DN-HDL for 24 hours, and cell proliferation was measured using Brdu assay. (Mean + SD, n = 6, *p<0.05; ***p<0.001; one-way ANOVA). Supplement Figure 2: Glycated high-density lipoprotein in vitro have a higher glycation level and a reduced capability to promote EC migration compared with normal and diabetic HDL. A: Normal HDL was incubated in 25 mM glucose solution at 37 °C over 7 days and HDL glycation level was measured using MS. B: HUVEC monolayers were scratched by manual scraping and treated with PBS, N-HDL, D-HDL, DN-HDL or G-HDL respectively at 100 ug/ml apoA-I for 10 hours. Migration into the wound was photographed (50X objective lens). Distance between gaps was measured, and the results were expressed as percentage of various HDL-treated cells in comparison with control. C: HUVECs were treated with PBS, N-HDL, D-HDL, DN-HDL or G-HDL at 100 ug/ml apoA-I for 10 hours respectively in transwell assay, and pictures were taken in 6 random high-power (50X) fields. Migratory cells were counted, and the results were expressed as percentage of various HDL-treated cells in comparison with control (*p<0.05; **p<0.01; ***p<0.001; one-way ANOVA). (DOCX 996 kb)

## Abbreviations

ApoA-I: apolipoprotein A-I
CKD: Chronic kidney disease
CVD: Cardiovascular disease
D-HDL: diabetic high-density lipoprotein
DN: diabetic nephropathy
DN-HDL: diabetic nephropathy high-density lipoprotein
N-HDL: normal high-density lipoprotein
