# Supplementary material for: HDL in diabetic nephropathy has less effect in endothelial repairing than diabetes without complications
Source: Lipids Health Dis. 2016 Apr 14;15:76. doi: 10.1186/s12944-016-0246-z (PMC4831084; doi:10.1186/s12944-016-0246-z)
Supplement: Additional file 1: — Supplement Figure 1: Diabetic and diabetic nephropathic high-density lipoprotein is less efficient in stimulating EC proliferation. HUVECs were treated with N-HDL, D-HDL and DN-HDL for 24 hours, and cell proliferation was measured using Brdu assay. (Mean + SD, n = 6, *p<0.05; ***p<0.001; one-way ANOVA). Supplement Figure 2: Glycated high-density lipoprotein in vitro have a higher glycation level and a reduced capability to promote EC migration compared with normal and diabetic HDL. A: Normal HDL was incubated in 25 mM glucose solution at 37 °C over 7 days and HDL glycation level was measured using MS. B: HUVEC monolayers were scratched by manual scraping and treated with PBS, N-HDL, D-HDL, DN-HDL or G-HDL respectively at 100 ug/ml apoA-I for 10 hours. Migration into the wound was photographed (50X objective lens). Distance between gaps was measured, and the results were expressed as percentage of various HDL-treated cells in comparison with control. C: HUVECs were treated with PBS, N-HDL, D-HDL, DN-HDL or G-HDL at 100 ug/ml apoA-I for 10 hours respectively in transwell assay, and pictures were taken in 6 random high-power (50X) fields. Migratory cells were counted, and the results were expressed as percentage of various HDL-treated cells in comparison with control (*p<0.05; **p<0.01; ***p<0.001; one-way ANOVA). (DOCX 996 kb) [file 12944_2016_246_MOESM1_ESM.docx]

**
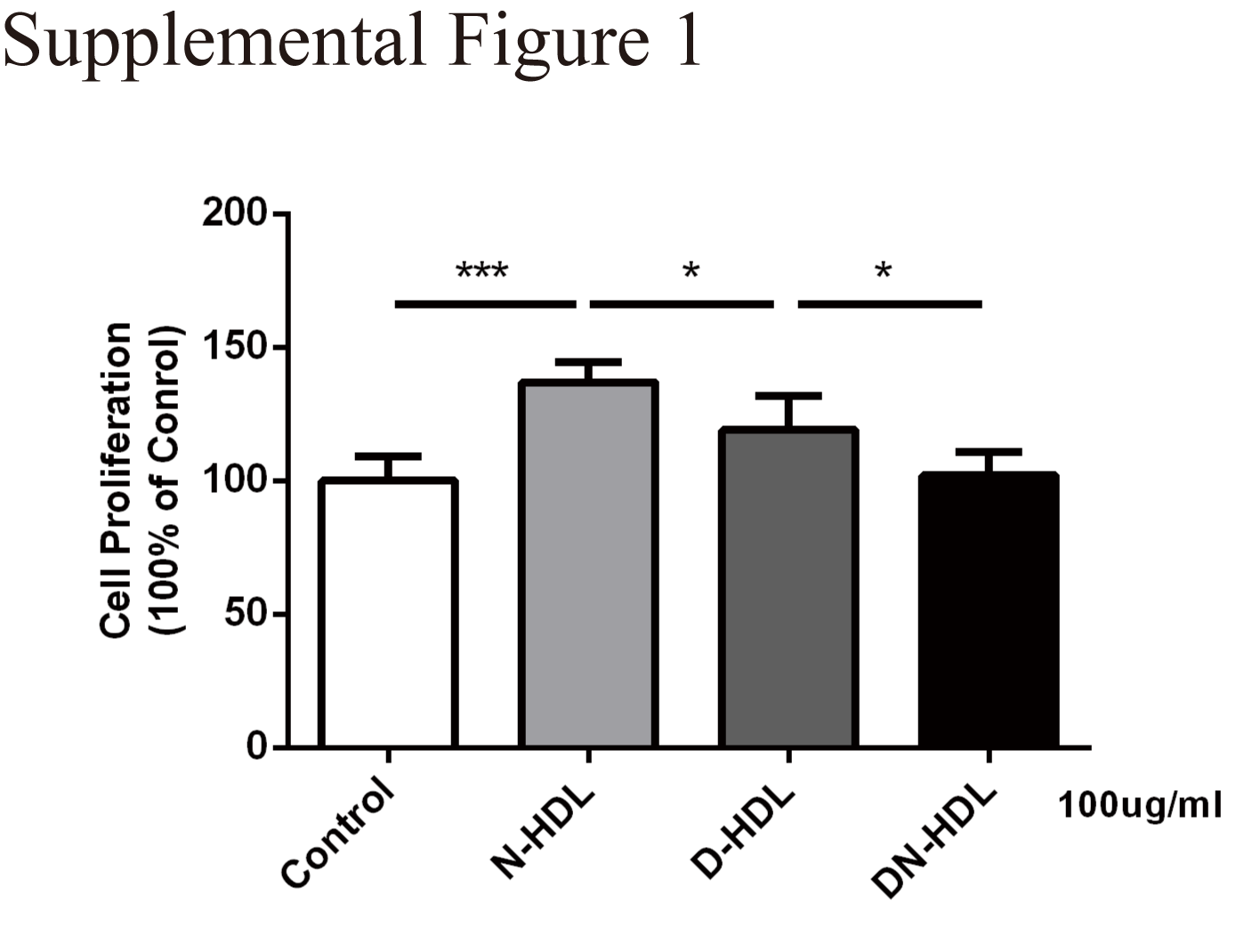
**

**Supplemental Figure 1: Diabetic and diabetic nephropathic high-density lipoprotein is less efficient in stimulating EC proliferation.**

HUVECs were treated with N-HDL, D-HDL and DN-HDL for 24 hours, and cell proliferation was measured using Brdu assay. (Mean + SD, n = 6, *p<0.05; ***p<0.001; one-way ANOVA)

**
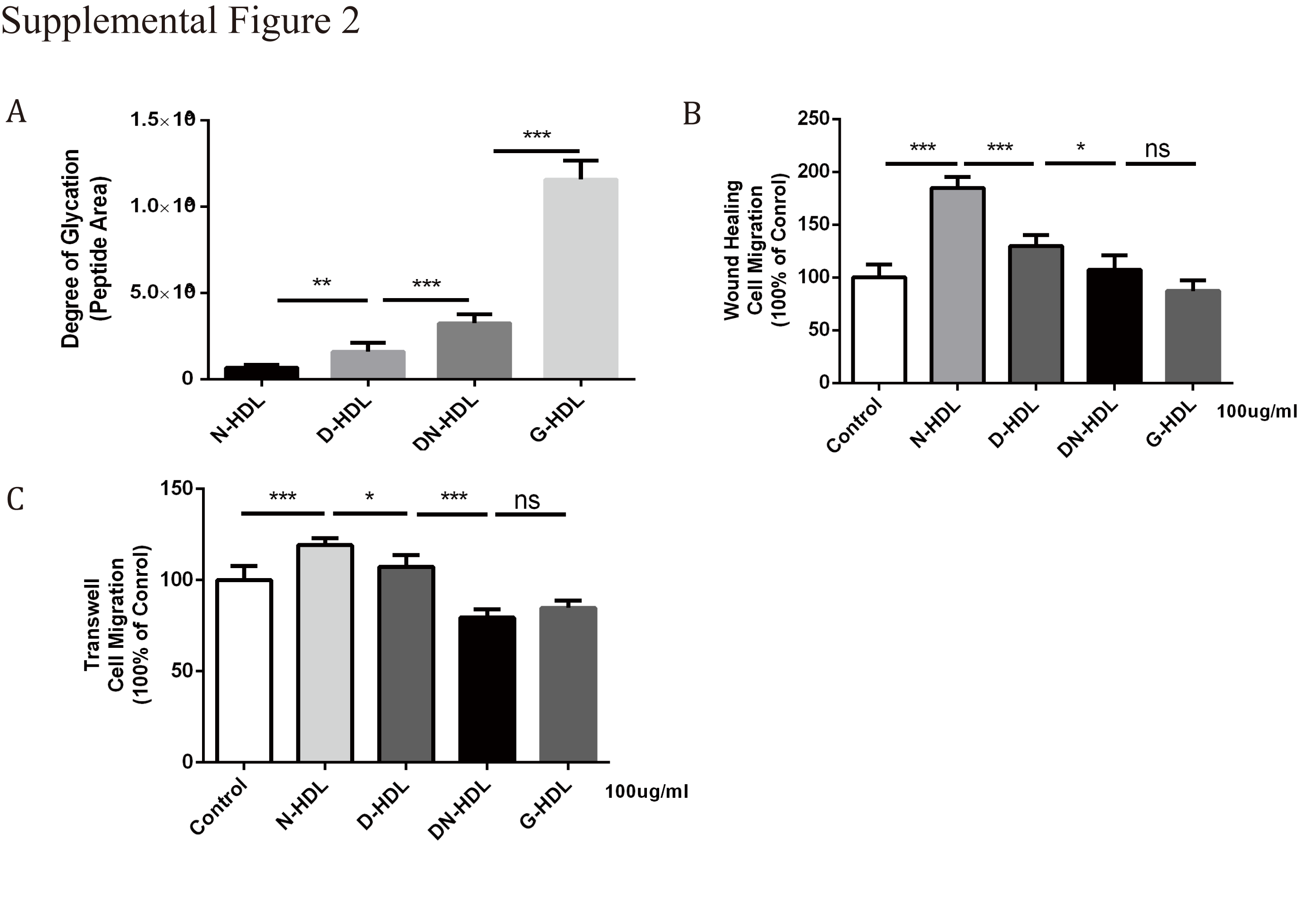
**

**Supplement Figure 2: Glycated high-density lipoprotein in vitro have a higher glycation level and a reduced capability to promote EC migration compared with normal and diabetic HDL.**

A: Normal HDL was incubated in 25mM glucose solution at 37℃ over 7 days and HDL glycation level was measured using MS. B: HUVEC monolayers were scratched by manual scraping and treated with PBS, N-HDL, D-HDL, DN-HDL or G-HDL respectively at 100ug/ml apoA-I for 10 hours. Migration into the wound was photographed (50X objective lens). Distance between gaps was measured, and the results were expressed as percentage of various HDL-treated cells in comparison with control. C: HUVECs were treated with PBS, N-HDL, D-HDL, DN-HDL or G-HDL at 100ug/ml apoA-I for 10 hours respectively in transwell assay, and pictures were taken in 6 random high-power (50X) fields. Migratory cells were counted, and the results were expressed as percentage of various HDL-treated cells in comparison with control (*p<0.05;**p<0.01; ***p<0.001; one-way ANOVA).
